# What Factors Influence Language Impairment? Considering Resilience as well as Risk

**DOI:** 10.1159/000444750

**Published:** 2016-05-03

**Authors:** Gina Conti-Ramsden, Kevin Durkin

**Affiliations:** ^a^The University of Manchester, Manchester, UK; ^b^School of Psychological Sciences and Health, University of Strathclyde, Glasgow, UK

**Keywords:** Language impairment, Resilience, Risk factors

## Abstract

The considerable variation observed in the profiles of children with language impairment (LI) raises challenges for the diagnosis, treatment and prevention of language difficulties, in particular since LI can present substantial issues calling for the investment of clinical, educational and public health resources. In this review paper, we examine biological, psychological and environmental factors that appear to influence the developmental course of LI. In this review paper we are interested not only in examining deficits and risk factors but also in identifying strengths of children with LI that can act as protective factors providing the child with a scaffold for more positive development and better outcomes.

## What Factors Influence Language Impairment? Considering Resilience as well as Risk

Children with language impairment (LI) have deficits in the ability to learn and use language (expressive and/or receptive) despite otherwise normal development. Approximately 7% of children are affected by LI [1]. This means that, on current UK birth rates, every year over 56,000 children start school with language difficulties.

Although LI has been conceptualized as a relatively ‘pure’ disorder of language, the condition is quite heterogeneous [2]. Research indicates developmental interactions between LI and difficulties acquiring literacy skills and more general non-verbal abilities throughout middle childhood, adolescence and beyond [3,4,5]. Children with language difficulties are at risk of less successful developmental and educational outcomes [6,7,8,9]. These children are more vulnerable to academic failure, social exclusion, behavioural and emotional difficulties, and to being bullied [5,10,11]. Yet negative sequelae are not inevitable. Some individuals achieve positive outcomes in a number of areas of functioning.

The considerable variation observed in LI raises challenges for the diagnosis, treatment and prevention of language difficulties, in particular since LI can present substantial issues calling for the investment of clinical, educational and public health resources. To date, much of the scientific effort focussed on LI has been on deficits and analysis of risk factors. In this paper, we also focus on resilience. We are interested in identifying strengths of children with LI that can act as protective factors providing the child with a scaffold for more positive development and better outcomes. In this paper we examine biological, psychological and environmental factors that appear to influence the developmental course of LI.

## Terminological Debate

Although LI is recognized internationally [12], professionals and academics working in speech and language therapy, psychology and education have struggled to find a common language to refer to these children. Currently, we do not have an agreed label that fosters information exchange and collaboration across disciplines and across different stages of children's development. Labels include ‘specific language impairment’, ‘language disorders’, ‘speech, language and communication needs’, ‘developmental language delay’ and ‘primary language difficulties’, and the list could go on. In addition, across the English-speaking world, there is variation both within and between countries as to how LI should be diagnosed [13,14]. The good news is that a multinational, multidisciplinary effort is currently underway to develop a diagnostic and terminological consensus within the field of LI [15]. In this article, we use the term ‘language impairment’.

## Children with LI

Typically, LI comes to the attention of clinicians as a result of concern from significant others about the child's progress with language learning. Children who develop LI are usually characterized by having language difficulties from the outset of the language-learning process. Instead of reaching developmental language milestones on schedule (first words around a child's first birthday, word combinations around the child's second birthday), most children with LI are slow from the beginning. It is a hallmark of LI that the majority of these children are late talkers: they are late in acquiring their first words and in putting together their first word combinations. It is not the case that children with LI start developing language normally and then stop and become delayed or lose what they have learned. Occurrence of ‘language loss’ in infancy is reported in some children with autism spectrum disorders but *not* in children with specific LI. This appears to be a distinguishing feature between the two disorders [16] and hence can be particularly useful for the differential diagnosis between specific LI and autism spectrum disorders in the preschool period. In the preschool and early childhood period, difficulties with the sound system of the language, i.e., phonology, can co-occur with LI but are not considered to be a hallmark of the disorder. By middle childhood, problems with sound production are usually resolved or less evident (unless there is orofacial motor difficulty/apraxia) and most children with LI are intelligible. It is also worth noting that a minority (5%) of children with LI are not late talkers [17]. These children can develop problems late, after having acquired single words. For these children, word combinations pose the biggest challenge in the trajectory of their language difficulties.

## What Factors Influence LI?

**Fig. 1:**
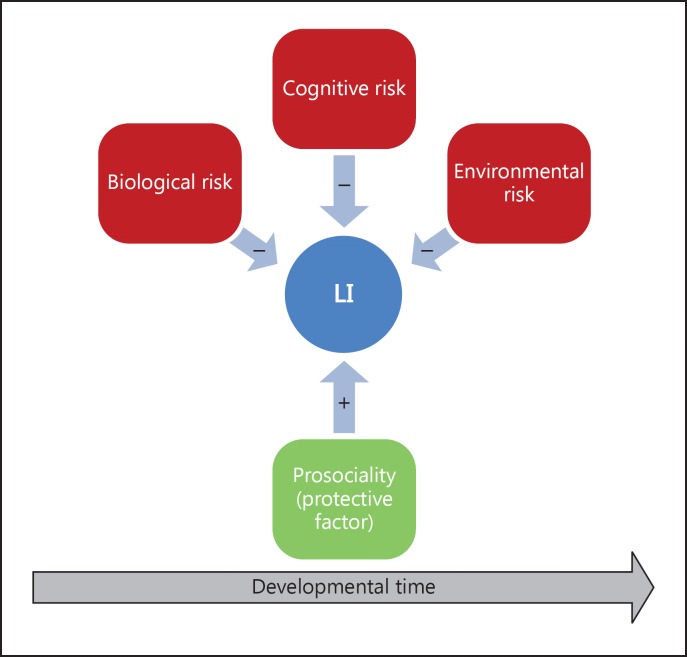
Risk and protective factors in LI.

It has become clear that LI is not the result of a single risk factor. A number of theories have been put forward, each focusing on a particular feature or set of characteristics, all of which have received some empirical support. It seems likely that multiple risk factors are implicated, and we argue that these can interact with protective factors to exacerbate or ameliorate LI. In prevention and intervention of LI it is important to examine strengths as well as difficulties (fig. 1).

### Biological Risk: Gender and Genetic and Neurobiological Factors

LI is more common in boys than in girls. The ratio of males to females is approximately 2:1 [for a review see [18]] and can be higher in samples from specialized settings such as language units [3:1; see [19]] and language schools [5:1; see [20]]. However, large epidemiological studies such as that by Tomblin et al. [1] have found approximately equal proportions of males and females with LI. Why may this be the case? What prompts parents/teachers to refer boys to services (and under-refer girls)? There is evidence that boys with LI are more likely than girls to have associated behavioural problems, ‘acting out’ their frustrations [for a review see [21]]. There is also some evidence to suggest that boys with language comprehension problems are more likely to exhibit aggression and externalizing problems [22,23]. Looking behind behaviour is crucial (see the RALLI video clip; https://www.youtube.com/watch?v = WTySmn_-X80), as is a detailed assessment of language comprehension [24] with appropriate targeted interventions (see, e.g., Contextualized Language Intervention strategies [25,26]).

There is strong evidence - from family aggregation studies, twin studies, adoption studies, pedigree analyses and genetic linkage analyses - that LI runs in families. The majority of children with LI have a family history of language difficulties, with a first-degree relative usually affected. The contribution of genetic factors is most clearly indicated in twin studies, such that identical twins have a higher concordance for LI than non-identical twins [27]. Patterns of inheritance appear to be complex, involving interactions between multiple genes [28]. We do know, however, that siblings of affected children are at a higher risk. On average, 30% of siblings develop LI [29]. These data on sibling genetic liability provide crucial information with regard to prevention. However, in reality limited resources are a barrier to action. How many services do we know that provide vigilant developmental assessments of siblings of children with LI?

Technological advances have made it possible to examine brain development in children with LI. However, few atypicalities have been identified. The most consistent neuroimaging findings suggest leftward asymmetry and an atypical cerebral volume. Electrophysiological evidence suggests abnormal auditory processing [30,31]. However, these abnormalities have also been observed in other developmental disorders. Thus, further research is needed to identify distinctive features of brain development in individuals with LI. As yet we do not have neurobiological markers for LI.

### Cognitive Risks: Memory Limitations

Different approaches emphasize risk factors in relation to deficits in different systems. Memory, information processing and temporal auditory processing mechanisms involved in the representation of grammar have all been proposed as influential risk factors for LI. In this article we focus on memory risk factors, in particular phonological short-term memory and procedural memory impairments in LI.

Research into the short-term memory capacity of children with LI has used Baddeley's model of working memory [32,33]. This model conceptualizes phonological short-term memory (PSTM) as a domain-specific area for the temporary storage of verbal information.

Gathercole and Baddeley [34,35] were among the first to demonstrate that non-word repetition, a measure of phonological short-term memory, was a fairly reliable risk factor for LI, as it discriminated between children with LI and either age- or language-matched, typically developing peers. Non-word repetition abilities have also been found to be heritable, as evidenced by twin and family studies involving children with LI [28,36].

Measures of PSTM, particularly as indexed by non-word repetition abilities, have since been widely used in research on children with LI. The majority of studies have involved school-age children [37,38,39,40], and available tools for measuring PSTM have also focused on children over 4 years of age [41]. However, Chiat and Roy [42,43] studied non-word and word repetition abilities in children as young as 2 years of age. They found that early difficulties with phonological processing and memory, as indexed by non-word and word repetition at 2-3 years of age, were not only correlated with concurrent language difficulties but were also predictive of language problems 2 years later (at 4-5 years of age). These authors have developed a clinical instrument, the Early Repetition Battery (ERB) [44], that provides a tool for the assessment of PSTM abilities in children as young as 2 years of age. Despite the availability of the ERB and other such instruments, PSTM is not yet routinely part of clinical assessment. The evidence indicates that it is time to include PSTM in the assessment of LI.

It is also worth noting that processing-dependent tasks such as non-word repetition have more validity across different languages [45,46,47,48]. In the assessment of multilingual children and children with differing backgrounds - for example, children of migrant families who do not speak, or are less proficient in, the majority language - the assessment of memory processes provides a promising tool for differentiating risk of LI from linguistic differences attributable to experiential factors.

In terms of long-term memory, it has been proposed that the grammatical impairments in children with LI are primarily caused by deficits in the procedural memory system [49]. The procedural memory system underlies the implicit learning, storage and retrieval of skills and knowledge [50,51]. Learning via the procedural memory system is often slow, with repetition or repeated exposures to the information required in order for a skill or knowledge to be learned [52], for example, learning grammatical morphemes such as past tense ‘-ed’ in English. Once information has been acquired, though, new knowledge and skills may be used without awareness. The learning and retrieval of information from the procedural memory system is said to be implicit. There is evidence that procedural memory deficits are a risk factor for LI.

A number of studies have found procedural learning is impaired in children with LI, even when the stimuli are non-verbal in nature [53,54,55]. To our knowledge, however, the assessment of procedural learning in children with LI is limited to research contexts, given the complexities of evaluating implicit processes. Further translational research is needed to develop tools which are usable in clinical contexts.

### Environmental Risk: Parental Education and Socioeconomic Status

Children are part of families and families are complex systems [56]. Children grow up in homes and social environments which can vary considerably in terms of parental practices and beliefs (associated with parental education) as well as access to experiences and the material worlds such as toys to play with or books to read (socioeconomic status). In general, samples of children with LI contain disproportionately high numbers of individuals from socioeconomically disadvantaged backgrounds [57,58]. Children living in poverty show language delays of 2 or more years by school entry [59].

One conceptualization of disadvantage has focused on children's linguistic input. Children of ‘professional’ parents who are more educated hear approximately 3 times more oral language than children of parents with lower education levels [60,61]. It is important to note, however, that there is little convincing evidence to support the claim that inadequate linguistic input (amount of parental talk, or use of child-directed speech) contributes to LI [18,62]. We know from cross-cultural research that language development is robust to variation in the amount and type of linguistic input needed for learning a native language. In Samoa and Papua New Guinea, for example, adults speak to children as they speak to adults - and much less frequently than parents of children in Western cultures - and children acquire language at the same pace as elsewhere in the world [63].

The regular linguistic environment of children with LI is simply not sufficient to overcome their language difficulties [64]. Children with LI require specialist input (from speech and language therapists or teachers). They need rich, scaffolded linguistic environments where specific aspects of language are targeted, highlighted, clarified and practiced to match the child's needs [65]. This evidence implies the need for appropriate language assessment of preschool children from families of low socioeconomic status, increased availability of speech and language therapy services throughout the school years as well as speech and language therapy input and teacher-therapist joint working in the classroom [59].

### Protective Factors: Sociability and Prosociality

Children with LI are sociable. Unlike children with autism spectrum disorders, those with LI want to interact socially [66]. In addition, they bring positive ‘prosocial’ attributes to interactions. Prosociality involves behaviours that are responsive to others’ needs and welfare, such as being helpful and sharing, showing kindness and consideration, cooperating with others and expressing empathy [67]. Research on LI and prosociality is only just emerging. Our own longitudinal work indicates that children with LI are moderately to highly prosocial and that prosociality confers developmental protection for these children [68].

Prosociality contributes to positive peer and social relationships in LI as well as emotional adjustment [9,69]. For example, participation in prosocial peer relationships appears to provide support for children who have negative experiences (such as bullying), facilitating coping and psychosocial resilience [70]. Prosocial adolescents are also reported to be less likely to engage in antisocial behaviours [71].

How often do we assess the level of prosociality in children with LI? How often do we monitor its developmental progress? What do we do to build on it? We believe it is crucial to identify and, if appropriate, further develop strengths of children with LI. The Strengths and Difficulties Questionnaire (SDQ) [72] is a freely available questionnaire that can be used by therapists, teachers, parents and also children and young people themselves. The SDQ has been used extensively in psychological research and has norms for children 3-16 years of age. The dedicated website provides downloadable materials and information (http://www.sdqinfo.com/).

To our knowledge there has not been a systematic effort to build on the prosocial tendencies of children with LI in intervention programmes. It is more common to target areas of deficits rather than strengths. A contrasting example is intervention efforts with children with autism spectrum disorders. There is an abundance of programmes that target improving the social skills and prosocial behaviours of children with autism spectrum disorders, although the effectiveness of such interventions has been limited [73]. It may well be time to re-think intervention goals for children with LI that include developing existing strengths that can in turn influence longer-term outcomes [see also [7]].

## Concluding Remarks

Risk and protective factors are crucial in the prevention and identification of LI. In addition to providing evidence-based information to parents who are concerned about their child, early identification of risk and protective factors affords the opportunity for targeted interventions. Language intervention has the potential to change the developmental course of children's language difficulties and improve long-term outcomes. Evidence suggests that there is fluidity in the rate of language growth in the preschool and early school years; some young children with LI experience accelerated growth during this early period of development [74]. The available literature also suggests that language continues to develop in this population with intervention. Older children and young people with LI continue to learn language at a steady pace beyond the early school years [3]. The above considerations - coupled with evidence of the efficacy of speech and language therapy treatment, particularly for interventions of longer duration [75,76] - make a strong argument for a risk-resilience developmental approach to prevention, identification and intervention in children with LI.

## Disclosure Statement

We declare no conflict of interests.
